# Comparison of the prognosis among in-hospital survivors of cardiogenic shock based on etiology: AMI and Non-AMI

**DOI:** 10.1186/s13613-024-01305-2

**Published:** 2024-05-12

**Authors:** Shih-Chieh Chien, Cheng-An Wang, Hung-Yi Liu, Chao-Feng Lin, Chun-Yao Huang, Li-Nien Chien

**Affiliations:** 1 Cardiovascular Division, Department of Internal Medicine, MacKay Memorial Hospital, Taipei, Taiwan; 2 Division of Cardiology, Department of Internal Medicine, Taipei Medical University Wan Fang Hospital, Taipei, Taiwan; 3 Health and Clinical Research Data Center, Office of Data Center, Taipei Medical University, Taipei, Taiwan; 4 Division of Cardiology and Cardiovascular Research Center, Department of Internal Medicine, Taipei Medical University Hospital, Taipei, Taiwan; 5 Institute of Health and Welfare Policy, College of Medicine, National Yang Ming Chiao Tung University, Taipei, Taiwan; 6 Graduate Institute of Data Science, College of Management, Taipei Medical University, Taipei, Taiwan

**Keywords:** Cardiogenic shock, Mortality, Rehospitalization, Long-term prognosis, Myocardial infarction

## Abstract

**Background:**

Current data on post-discharge mortality and rehospitalization is still insufficient among in-hospital survivors of cardiogenic shock (CS), including acute myocardial infarction (AMI) and non-AMI survivors.

**Methods:**

Patients with CS who survived after hospital discharge were selected from the Taiwan National Health Insurance Research Database. Each patient was followed up at 3-year intervals. Mortality and rehospitalization were analyzed using Kaplan-Meier curves and Cox regression models.

**Results:**

There were 16,582 eligible patients. Of these, 42.4% and 57.6% were AMI-CS and non-AMI-CS survivors, respectively. The overall mortality and rehospitalization rates were considerably high, with reports of 7.0% and 22.1% at 30 days, 24.5% and 58.2% at 1 year, and 38.9% and 73.0% at 3 years, respectively, among in-hospital CS survivors. Cardiovascular (CV) problems caused approximately 40% mortality and 60% rehospitalization. Overall, the non-AMI-CS group had a higher mortality burden than the AMI-CS group owing to older age and a higher prevalence of comorbidities. In multivariable models, the non-AMI-CS group exhibited a lower risk of all-cause mortality (adjusted hazard ratio [aHR] 0.69, 95% confidence interval [CI] 0.60 to 0.78) and CV mortality (aHR 0.65, 95% CI 0.54 to 0.78) compared to the AMI-CS group. However, these risks diminished and even reversed after one year (aHR 1.13, 95% CI 1.03 to 1.25 for all-cause mortality; aHR 1.27, 95% CI 1.09 to 1.49 for CV mortality).This reversal was not observed in all-cause and CV rehospitalization. For rehospitalization, AMI-CS was associated with the risk of CV rehospitalization in the entire observation period (aHR:0.80, 95% CI:0.76–0.84).

**Conclusions:**

In-hospital AMI-CS survivors had an increased risk of CV rehospitalization and 30-day mortality, whereas those with non-AMI-CS had a greater mortality risk after 1-year follow-up.

**Supplementary Information:**

The online version contains supplementary material available at 10.1186/s13613-024-01305-2.

## Introduction

Reports from several countries suggest a rising burden of cardiogenic shock (CS) because of its steadily rising incidence and persistently growing expenditure [1–4]. Patients with CS develop severe circulatory system derangement and acute organ hypoperfusion. Reversing this critical condition requires multidisciplinary collaboration to provide sufficient circulatory and organ support, relieve myocardial ischemia, and limit complications. Recent CS studies have demonstrated modest improvements in prognosis during hospitalization [2–4]. This implies that more patients survive to be discharged and face another challenge in the post-acute period [2]. Indeed, some acute myocardial infarction (AMI) studies have suggested that patients with CS remained in the high-risk population for mortality and rehospitalization in subacute periods of approximately 30 days to 1 year [5–7]. This highlights the importance of understanding the features of short- and long-term prognoses in all patients with CS.

Post-acute management should be tailored individually by exploring the clinical and prognostic characteristics of the CS subgroups. For example, the clinical features of CS change depending on the etiology of AMI or non-AMI. In the past, AMI was thought to be the major cause of CS and was associated with a higher risk of in-hospital mortality [8–10]. Contemporary trends have demonstrated an increasing proportion of non-AMI-associated CS (non-AMI-CS) versus AMI complicated by CS (AMI-CS), and non-AMI is becoming the leading cause of CS in some regions [2, 11]. More therapeutic innovations with evidence in AMI-CS than in non-AMI-CS in the modern era reflect a more predominant survival improvement in AMI-CS [2]. Consequently, it is important to understand the differences in short- and long-term discharge outcomes between AMI-CS and non-AMI-CS to develop a clinical strategy accordingly.

We performed a nationwide cohort study of AMI and non-AMI in-hospital CS survivors using the National Health Insurance Research Database (NHIRD). We aimed to study short- and long-term mortality and rehospitalization in overall and subgroups of patients with CS based on the etiology.

## Methods

### Data source and study population

The study design was modified and extended from a published study, Cardiogenic Shock in Taiwan (CSiT) [2]. Data were extracted from the NHIRD in Taiwan. The NHIRD includes complete data on outpatient visits, hospital admissions, medication prescriptions, disease diagnoses, medical procedures, and vital statuses of 99% of Taiwan’s population. Diagnoses in the NHIRD have been coded according to the International Classification of Diseases, Ninth Revision, Clinical Modification (ICD-9-CM) codes and ICD-10-CM since 2016. The NHIRD can be linked to the National Death Registry (NDR) by using the unique encrypted identification of each beneficiary. The accuracies of the NHIRD and NDR have been analyzed in previous studies [12, 13]. The study protocol was approved by the Institutional Review Board of MacKay Memorial Hospital (23MMHIS201e).

Our data period was between 2010 and 2020. To ensure patients could complete a three-year follow-up, individuals were selected if they had a diagnostic code for CS (ICD-9-CM of 785.51 and ICD-10-CM of R57.0) based on inpatient claims between January 1, 2011, and December 31, 2017, and survived hospital discharge. The exclusion criteria were the same as those in the previous study, including (1) no age or sex information (*n* = 60), (2) age < 18 years (*n* = 332), and (3) length of hospital stay > 365 days (*n* = 8). The last exclusion criterion remained because the rationale for a long hospital stay was uncertain. Patients who had a death record within two days after the date of hospital discharge were also excluded because of possible impending death discharge. We excluded patients with CS before 2011 to prevent the potential influence of the national AMI accreditation policy implemented in 2009 in the study region on mortality [2].

### Mortality and rehospitalization

Death records were obtained from the NDR, a nationwide registry for the causes of death. In addition to all-cause mortality or rehospitalization, we assessed the impact of cardiovascular (CV) disease on outcomes and defined CV mortality and CV rehospitalization. The first ICD code assessed the primary cause of death.

### Covariables

Most comorbidities observed in the current study, including hypertension, dyslipidemia, coronary artery disease, prior myocardial infarction (MI), renal failure, congestive heart failure, peripheral arterial disease, and atrial fibrillation, were associated with mortality. Patients were considered to have a specific condition if they had at least two diagnostic claims made during outpatient visits or one during hospital admission before the index date of the CS. Renal failure was identified by the National Catastrophic Illness Patient Registry, which provides substantial benefits regarding medical expenses. Medical procedures performed at CS admission were considered, including percutaneous coronary intervention (PCI); coronary artery bypass graft (CABG) and heart transplantation; and mechanical circulatory support (MCS) devices using intra-aortic balloon pumps (IABPs), extracorporeal membrane oxygenation (ECMO), and ventricular assist devices (VADs). Medications including angiotensin converting enzyme inhibitors/angiotensin II receptor antagonists (ACEI/ARB) and beta-blocker prescribed at the first outpatient clinic visit were evaluated and recorded. According to the medical capabilities, hospitals were divided into three levels (medical center, regional hospital and district hospital) and hospital levels were accredited regularly by the central health authorities, namely the Joint Commission of Taiwan and the Ministry of Health and Welfare [14]. In Taiwan, official regulations adhere to hospice palliative care guidelines established by the “Hospice Palliative Care Act” in 2000. The medical costs for hospice palliative mode have been covered by National Health Insurance since September 2009, and relevant records after discharge were assessed. Details of the disease diagnosis and treatment procedure codes were presented in Additional file 1.

### Statistical analysis

We compared demographics, healthcare resource use, comorbidities, hospital level, and year of CS diagnosis between the two study cohorts (AMI vs. non-AMI). Quantitative variables are expressed as mean, and standard deviation, and qualitative variables are presented as absolute frequencies (number of patients) and relative frequencies (percentages). Two-sample t-tests were used to compare continuous variables between groups. The index hospital discharge date was the first day of follow-up. We used landmark analysis to show the time-varying prognosis of mortality and rehospitalization at 30 days and 1-year point since the prognostic impact of AMI-CS (or non-AMI-CS) was inconsistent. Those choices were informed by both data-driven selection, which involved identifying exponential curves that best fit the observed survival curve and including commonly used time points in the literature. Event-free survival was calculated using the Kaplan–Meier method. The hazard ratio (HR) for the Cox proportional hazards regression model was used along with the corresponding standard error, 95% confidence interval (CI), and p value. Baseline demographics, comorbidities, hospital level, index year, medications and cardiopulmonary resuscitation were included in the multivariable adjustment. AMI condition and cardiac procedures, including PCI, CABG, or heart transplantation, were not included for adjustment owing to the different disease pathological nature. We also excluded MCS for adjustment because of the inequalities in MCS use in the study region. As death is a competing event in evaluating the risk of rehospitalization, we performed a competing risk analysis and treated death as a competing risk. Statistical analyses were performed using SAS/STAT version 9.4 (SAS Institute, Cary, NC, USA). A p-value < 0.05 was considered statistically significant.

## Results

### Baseline characteristics

Between January 1, 2011, and December 31, 2017, 7,037 (42.4%) and 9,545 (57.6%) patients were AMI-CS and non-AMI-CS in-hospital survivors, respectively, and were followed up for 3 years. The prevalence of CS survivors after discharge gradually increased during the observation period (p for trend < 0.001) (Additional File 2). The baseline characteristics of the patients are shown in Table 1. Compared to patients with AMI-CS, those with non-AMI-CS were older and more likely to be female. They had a higher prevalence of congestive heart failure, hypertension, renal failure, stroke, malignancy, and atrial fibrillation, whereas no significant differences were found in diabetes, peripheral arterial disease, or VAD implementation. A higher percentage of non-AMI-CS patients were not treated in medical centers and they received less medications of ACEI/ARB and beta-blocker after hospital discharge. However, non-AMI CS survivors experienced fewer cardiac arrests, coronary interventions (PCI and CABG), and mechanical support (IABP and ECMO). The heart transplantation rate was borderline higher in the non-AMI-CS group than that in the AMI-CS group (0.6% vs. 0.4%; *p* = 0.054). According to records, there were 19.3% in-hospital survivors had hospice palliative cares during the entire follow up. Of them, 32.5% were AMI-CS and 67.5% were non-AMI-CS (Additional File 3).

**Table 1:** Baseline characteristics according to CS etiology: AMI and non-AMI

	AMI	Non-AMI	*P*
Overall	*N* = 7,037	*N* = 9,545	
**Demographics**			
Age (years) mean ± SD	65.9 ± 13.7	68.9 ± 16.4	< 0.001
Male sex (%)	5372 (76.3%)	5380 (56.4%)	< 0.001
**Comorbidities**			
Congestive heart failure	2351 (33.4%)	4587 (48.1%)	< 0.001
Hypertension	3199 (45.5%)	5213 (54.6%)	< 0.001
Diabetes mellitus	2442 (34.7%)	3215 (33.7%)	0.171
Peripheral arterial disease	85 (1.2%)	138 (1.4%)	0.189
Dyslipidemia	1885 (26.8%)	1664 (17.4%)	< 0.001
Coronary artery disease	5395 (76.7%)	3729 (39.1%)	< 0.001
Prior myocardial infarction	939 (13.3%)	721 (7.6%)	< 0.001
Renal failure	446 (6.3%)	959 (10.0%)	< 0.001
Stroke	790 (11.2%)	1596 (16.7%)	< 0.001
Malignancy	416 (5.9%)	999 (10.5%)	< 0.001
Atrial fibrillation	529 (7.5%)	2290 (24.0%)	< 0.001
**Hospital level**, ***n*(%)**			< 0.001
Medical center	2759 (39.2%)	3199 (33.5%)	
Regional hospital	3867 (55.0%)	4898 (51.3%)	
District hospital	411 (5.8%)	1448 (15.2%)	
**Index year**, ***n*(%)**			0.061
2011–2013	2853 (40.5%)	4037 (42.3%)	
2014–2016	3133 (44.5%)	4159 (43.6%)	
2017	1051 (14.9%)	1349 (14.1%)	
**CS conditions**, ***n*(%)**			
Cardiac arrest	1105 (15.7%)	1073 (11.2%)	< 0.001
STEMI	2952 (41.9%)	0	
NSTEMI	4085 (58.1%)	0	
**Cardiology procedure**, ***n*(%)**			
PCI	5297 (75.3%)	824 (8.6%)	< 0.001
CABG	629 (8.9%)	255 (2.7%)	< 0.001
Heart transplantation	26 (0.4%)	55 (0.6%)	0.059
**Mechanical support**, ***n*(%)**			
IABP	3084 (43.8%)	909 (9.5%)	< 0.001
ECMO	511 (7.3%)	452 (4.7%)	< 0.001
VAD	27 (0.4%)	45 (0.5%)	0.396
**Medication**, ***n*(%)**			
ACEI/ARB	2343 (33.3%)	1884 (19.7%)	< 0.001
Beta-blocker	2617 (37.2%)	1898 (19.9%)	< 0.001

*Abbreviation*: CABG: coronary artery bypass graft, CS: cardiogenic shock, ECMO: extracorporeal membrane oxygenation, IABP: intra-aortic balloon pump, NSTEMI: non-ST-segment elevation myocardial infarction, PCI: percutaneous coronary intervention, SD: standard deviation, STEMI: ST-segment elevation myocardial infarction, VAD: ventricular assist device. ACEI: angiotensin converting enzyme inhibitors, ARB: angiotensin II receptor antagonist

### Mortality

During the 3-year follow-up period, there were 6,449 deaths, accounting for 38.9% of the entire cohort, and 43.0% were due to CV etiologies (Table 2, Additional File 4). The mortality rates within 30 days, 1 year, and 3 years were 1,162 (7.0%), 4,062 (24.5%), and 6,449 (38.9%), respectively, for all-cause mortality, and 624 (3.8%), 1,863 (11.2%), and 2,774 (16.7%), respectively, for only CV reasons.

**Table 2:** Mortality and rehospitalization according to CS etiology: AMI and non-AMI

	All		AMI	Non-AMI
Outcomes/time intervals	*N* = 16,582		*N* = 7,037	*N* = 9,545
All-cause mortality				
Within 30 d	1162(7.0%)		457(6.5%)	705(7.4%)
Within 1 y	4062(24.5%)		1461(20.8%)	2601(27.2%)
Within 3 y	6449(38.9%)		2258(32.1%)	4191(43.9%)
CV mortality				
Within 30 d	624(3.8%)		276(3.9%)	348(3.6%)
Within 1 y	1863(11.2%)		763(10.8%)	1100(11.5%)
Within 3 y	2774(16.7%)		1054(15%)	1720(18.0%)
All-cause rehospitalization				
Within 30 d	3668(22.1%)		1381(19.6%)	2287(24.0%)
Within 1 y	9651(58.2%)		4012(57.0%)	5639(59.1%)
Within 3 y	12,101(73.0%)		5032(71.5%)	7069(74.1%)
CV rehospitalization				
Within 30 d	2036(12.3%)		892(12.7%)	1144(12.0)
Within 1 y	6453(38.9%)		3057(43.4%)	3396(35.6%)
Within 3 y	8651(51.6%)		3955(56.2%)	4606(48.3%)

*Abbreviation*: AMI: acute myocardial infarction, CV: cardiovascular, d: days, y: years

**Fig. 1:**
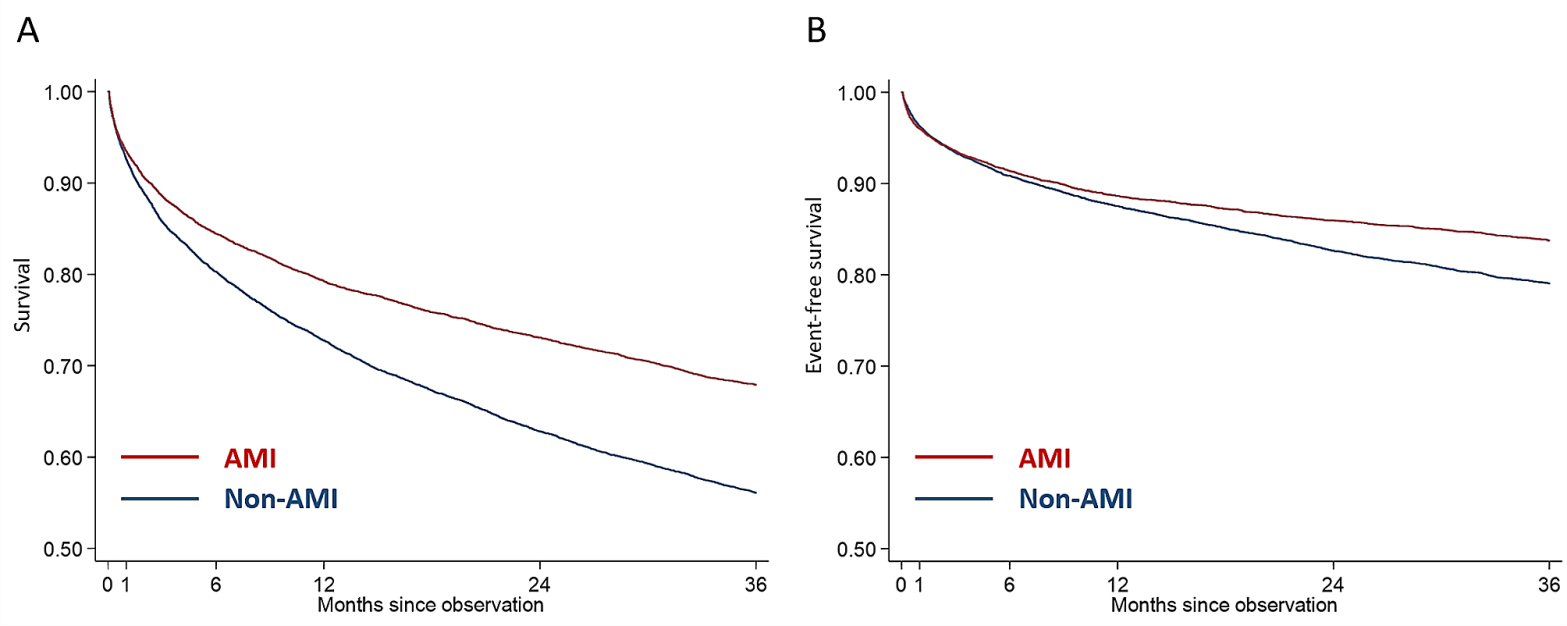
Three-year (A) all-cause and (B) cardiovascular mortality. Kaplan-Meier curves showed that patients of non-AMI-CS had higher all-cause and cardiovascular mortality compared with those of AMI-CS. Abbreviation: AMI: acute myocardial infarction, CS: cardiogenic shock

The Kaplan-Meier curves showed that the non-AMI-CS group was likely to have a more significant mortality burden based on total number and percentage than the AMI-CS group (Fig. 1A, B). The crude HRs at the three different follow-up intervals consistently showed a higher mortality risk (all-cause and CV) in the non-AMI-CS group than in the AMI-CS group. Based on landmark analysis, three-time intervals were identified: 1–30 days, 31–365 days, and 1–3 years. The adjusted mortality risks based on the CS etiology (AMI vs. non-AMI) appeared to vary throughout the follow-up time intervals. When considering the AMI-CS group as the reference, the adjusted hazard ratios (HRs) for all-cause mortality in the non-AMI-CS group were 0.69 (95% CI: 0.60–0.78) from 1 to 30 days, 1.00 (95% CI: 0.92–1.09) from 31 to 365 days, and 1.13 (95% CI: 1.03–1.25) from 1 to 3 years, indicating a reversal in risk after one year of follow-up. Similar patterns were observed for CV mortality (Table 3, Central Fig.).

**Table 3:** Crude and adjusted hazard ratios for mortality and rehospitalization according to CS etiology: AMI and non-AMI

	Event	cHR	95% CI	*P*	aHR^#^	95% CI	*P*
Outcomes/time intervals	*AMI (reference group) : non-AMI						
All-cause mortality							
Within 30d	457 : 705	1.14	1.01–1.28	0.029	0.69	0.60–0.78	< 0.001
From 31 to 365d	637 : 1176	1.46	1.35–1.58	< 0.001	1.00	0.92–1.09	0.973
From 1 to 3y	797 : 1590	1.69	1.55–1.84	< 0.001	1.13	1.03–1.25	0.011
CV mortality							
Within 30d	276 : 348	0.93	0.80–1.09	0.382	0.65	0.54–0.78	< 0.001
From 31 to 365d	311 : 478	1.20	1.07–1.34	0.002	0.92	0.81–1.04	0.189
From 1 to 3y	291 : 620	1.81	1.57–2.08	< 0.001	1.27	1.09–1.49	0.003
All-cause rehospitalization							
Within 30d	1381 : 2287	1.26	1.18–1.34	< 0.001	1.04	0.96–1.12	0.375
From 31 to 365d	1875 : 2363	0.95	1.05–0.99	0.497	0.86	0.81–0.91	< 0.001
From 1 to 3y	1020 : 1430	1.18	1.09–1.28	< 0.001	1.05	0.95–1.15	0.357
CV rehospitalization							
Within 30d	892 : 1144	0.95	0.87–1.03	0.231	0.87	0.79–0.97	0.009
From 31 to 365d	1512 : 1488	0.74	0.69–0.78	< 0.001	0.72	0.67–0.77	< 0.001
From 1 to 3y	898 : 1210	0.95	0.87–1.03	0.235	0.90	0.82–1.00	0.046

* AMI-CS as reference group

# Adjusted for the variables age, sex, comorbidities, hospital level, index year, medications

Competing risk analysis was used for rehospitalization

*Abbreviation*: CS: cardiogenic shock, AMI: acute myocardial infarction, aHR: adjusted hazard ratio, cHR: crude hazard ratio, CV: cardiovascular, CI: confidence interval, d: days, y: years

#### Rehospitalization

Rehospitalization was frequently observed in hospital survivors of CS, ranging from 22% within 30 days to > 70% within 3 years of follow-up. More than 50% of these were due to CV etiologies (Table 2). Rehospitalization cases within 30 days, 1 year, and 3 years were 3,668 (22.1%), 9,651 (58.2%), and 12,101 (73.0%), respectively, for all-cause, and 2,036 (12.3%), 6,453 (38.9%), 8,651 (51.6%), respectively, for only CV reasons (Table 2).

**Fig. 2:**
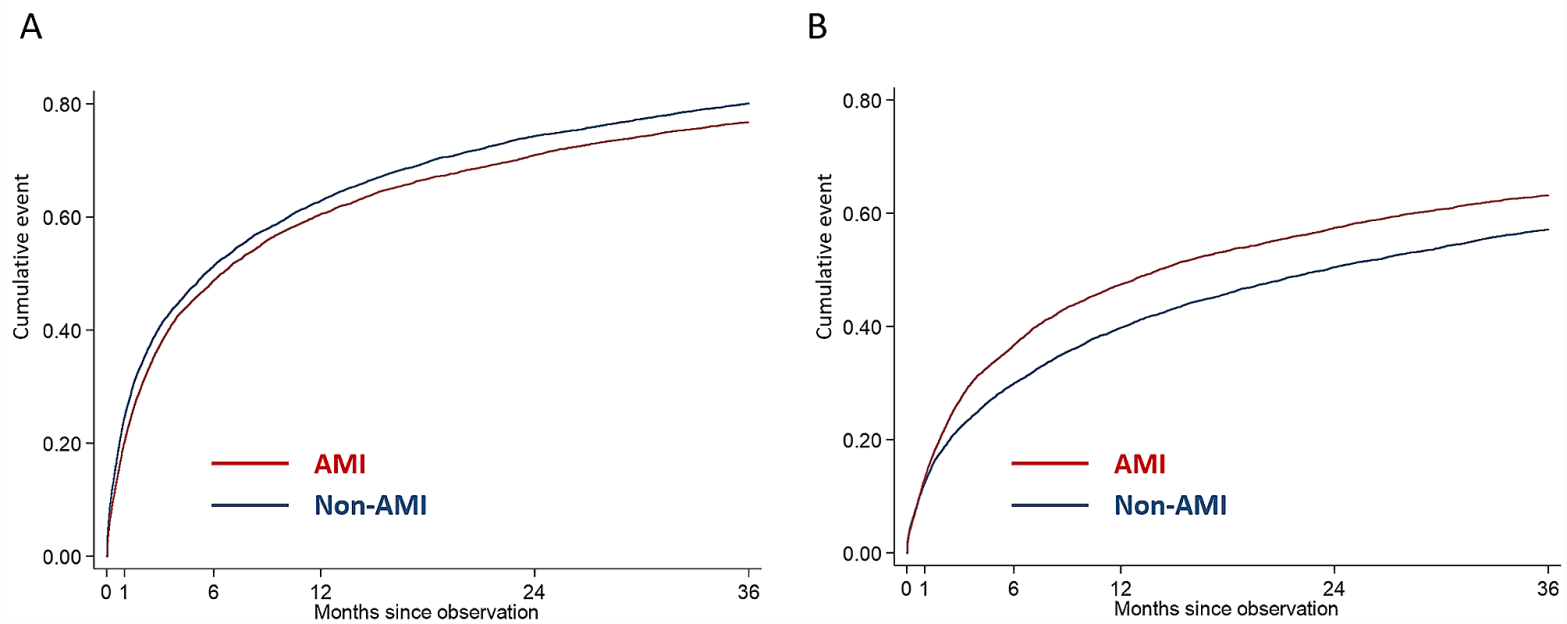
Three-year (A) all-cause and (B) cardiovascular rehospitalization. Kaplan-Meier curves showed that patients of non-AMI-CS had higher all-cause rehospitalization but lower cardiovascular rehospitalization compared with those of AMI-CS. Abbreviation: AMI: acute myocardial infarction, CS: cardiogenic shock

The non-AMI-CS group appeared a greater burden of all-cause rehospitalization compared to the AMI-CS group (Fig. 2A). However, after adjusting covariables, the non-AMI-CS group had a lower risk of all-cause rehospitalization from 31 to 365d (HRs:0.86) compared to AMI-CS group (Table 3).

In-hospital survivors of non-AMI-CS were associated with a lower risk of CV rehospitalization throughout the follow-up period (Table 2; Fig. 2B). Considering the AMI-CS as reference, the adjusted HRs for the non-AMI-CS group were 0.87 (95% CI: 0.79–0.97, *p* = 0.009) from 1 to 30 days, 0.72 (95% CI: 0.67–0.77, *p* < 0.001) from 31 to 365 days, and 0.90 (95% CI:0.82-1.00, *p* = 0.046) from 1 to 3 years, respectively (Table 3).

## Discussion

In this nationwide population-based study, we identified several clinical and prognostic issues among AMI and non-AMI in-hospital survivors of CS. First, both mortality and rehospitalization rates were high, increasing up to 24.5% and 38.9% within one and three years, respectively, for mortality and 58.2% and 73.0% within one and three years, respectively, for rehospitalization (Central Fig). Second, CV problems caused approximately 40% mortality and 60% rehospitalization. Third, in-hospital survivors with AMI-CS were associated with an increased risk of CV rehospitalization and mortality within 30 days (both all-cause and CV), whereas those with non-AMI-CS were at a higher risk of mortality after 1 year of follow-up (Central Fig).

### Demographic changes and comparisons: AMI vs. Non-AMI

Although CS is a life-threatening condition associated with high in-hospital mortality, recent reports have shown declining mortality trends, probably reflecting the current therapeutic advances [2–4]. Based on our data, improvements in in-hospital survival were observed in both AMI and non-AMI patients. However, to the best of our knowledge, current studies have mainly focused on in-hospital CS survivors with AMI etiology and provided information limited to long-term outcomes. Our study provides broader features of the local region involving both AMI and non-AMI patients. Owing to the nature of the disease, individuals who had non-AMI-CS and survived to be discharged tended to be older and had more complex comorbidities. Simultaneously, those patients also had a relatively lower percentage of medical center hospitalization and MCS use due to local health policy for AMI [2]. This reflects the gap between the reality and ideal care mode of CS patients despite the guidelines and consensus recommended to centralize all eligible patients in a medical hub to offer comprehensive, collaborative, and multidisciplinary care [15–17]. A well-organized regional system can optimize resource allocation and facilitate early treatment. Consequently, the quality of care and prognosis can be improved [15–18]. Despite potential underestimation, near one fifth in-hospital survivors of CS received palliative programs. This percentage was higher than another study [19]. It is urgent to explore the cost-effectiveness and quality improvement after integrating palliative care in CS patients.

### Mortality

Understanding specific reasons for mortality is essential for assessing the lifelong risks among in-hospital survival of CS and for designing a post-acute care model. With using first ICD code as the primary diagnosis, CV and respiratory reasons were the major causes of deaths during 3-year follow up (Additional File 4). However, it is imperative to exercise caution when interpreting these data due to the retrospective nature of the study design and the absence of chart reviews. Developing a well-defined prospective study to investigate the exact reason for death is ideal.

Overall, the non-AMI-CS group had a higher burden of mortality throughout the 3 years of follow-up. Our data are consistent with previous studies that showed that patients with AMI-CS were associated with a higher risk of 30-day mortality than those without AMI-CS after adjusting for baseline factors [9, 10]. The difference between crude and adjusted mortality could be partly explained by the increase in the percentage of “young patients with AMI” and the decline in the severity of AMI in recent years [20–22]. The lower severity of AMI was reflected in the lower overall mortality since individuals with non-AMI-CS were usually more fragile with complicated comorbidities, as shown in our data. Once the baseline differences were adjusted for, the AMI-CS group presented a higher risk of 30-day mortality than the non-AMI-CS group. Notably, the early mortality risk of AMI-CS attenuated gradually and was reversed after 1 year of follow-up. Landmark analysis showed that non-AMI-CS was an independent risk factor for all-cause and CV mortality in the time interval of 1–3 years. This finding supports the “AMI-CS storm” concept, which was once reported to last around half to 1 year [5, 6, 23]. Shah et al. investigated 112,668 AMI in-hospital survivors and found that those with AMI-CS (5%) were associated with higher all-cause mortality and rehospitalization in the first 60 days (adjusted HR:1.28; 95% CI:1.21 to 1.35) and the risk was similar thereafter (adjusted HR:0.95 for days 61 to 365; 95% CI:0.89 to 1.01) [5]. Aissaoui et al. also investigated an AMI registry comprising 99 shock and 3,312 non-shock patients. They found that patients with AMI-CS had a 2.8-times higher risk of mortality within 1 year than those without shock. However, the long-term (1–5 years) mortality risk did not differ between the two groups [6]. Hence, aggressive monitoring and intervention could be crucial and cost-effective for in-hospital survivors of AMI-CS during the subacute phase.

### Rehospitalization

Shah et al. and Mahmoud et al. have showed an exceptionally high risk of 30-day rehospitalization among in-hospital survivors of CS for the AMI subgroup based on the data from the US [7, 24]. Approximately one-fifth of our data are consistent with their reports. Moreover, we demonstrated similar and high rehospitalization rates that persistently increased to 58% within 1 year and 73% within 3 years in the non-AMI subgroup. CV causes contribute to 50–60% of all rehospitalizations, which is close to but higher than reports from the US readmission dataset. Current guidelines suggest that multidisciplinary and patient-centered care after hospital discharge helps lower rehospitalization rates among high-risk populations such as those with AMI and heart failure [15, 25]. Considering the similar or even higher risk among patients with CS, initiating a post-acute care program to protect this fragile population is worthy and critical. For instance, most patients who recover from an acute illness experience predominant muscle weakness and polyneuropathy, known as intensive care unit-acquired weakness [26]. Active implementation of a rehabilitation program significantly improves patients’ activities and quality of life [27]. Indeed, a Danish survey of survivors of out-of-hospital cardiac arrest demonstrated that a considerable number of individuals had chronic physical health impairments, as assessed by the SF-12, until 20 years of follow up [28]. Moreover, respiratory, infection, and gastrointestinal factors are considered other important etiologies for rehospitalization, accounting for approximately 10% each [7, 24]. This implies persistently impaired organ function and self-care abilities when patients with CS return home. Comprehensive organ function assessment and tailored treatment should be individualized during the outpatient periods [16].

### Clinical implication

Current risk scores for CS, such as CardShock, SAVE, modified SAVE, and ENCOURAGE, have mainly been proposed for short-term or in-hospital mortality and cannot be applied for long-term mortality [9, 29–31]. Some prognostic surrogates, such as AMI, seem to lose their ability to predict long-term mortality, as demonstrated in the present study. Moreover, valid prognostic scores for predicting rehospitalization remain lacking. Therefore, targeting different outcomes and follow-up periods is the next important task for improving risk discrimination.

During the recovery phase, in-hospital CS survivors frequently experience heart failure symptoms. Although several pharmacotherapies have shown clinical benefits in patients with heart failure, patients with CS have been excluded because of hemodynamic instability during index hospitalization [32, 33]. It is urgent to determine whether these promising medications are effective for both heart failure and post-acute care of CS.

### Study limitations

Our study has several limitations. First, it was a nationwide claims-based cohort study conducted in real-world settings. The accuracy of disease diagnosis was based on discharge claims, as a review article reported that the positive predictive value of most diseases was in the 80–99% range [34]. Clinically relevant imaging and laboratory data were unavailable. Second, the study was conducted in Taiwan and may not be generalizable to other populations due to various factors such as differences in resource allocation, policy frameworks, and ethnic considerations [35]. Certain policies and treatment patterns in different countries may have influenced these results. Thus, replication of study designs is warranted. Third, we acknowledged the inability to adjust for disease heterogeneity (e.g., disease severity). Indeed, attempting to classify diverse etiologies encompassing the entire non-AMI CS patient cohort solely based on disease codes (ICD-9 or 10) presents considerable challenges and may introduce biases. Fourth, because terminal discharge (going home to die) is a well-adopted tradition in Taiwan, we excluded patients who died within 3 days after the index discharge date, which might have underestimated short-term (1–30 day) mortality. Finally, we did not differentiate the exact etiology of mortality or rehospitalization because some overlaps and uncertainty of the major diagnostic coding existed in the claims database, which biased the categorization.

## Conclusions

The trend of in-hospital survivors with a diagnosis of CS is persistently increasing in Taiwan; non-AMI-CS plays an important role and accounts for nearly three-fifths of all cases. Compared with other high-risk diseases, mortality and rehospitalization among in-hospital survivors of CS are considerably high, with reports of 38.9% and 73.0% in 3 years, respectively, and CV problems remain a major cause. AMI-CS has time-varying prognostic effects. Patients with AMI-CS were associated with higher 30-day mortality, while such associations were attenuated and even reversed after a 1-year follow-up, suggesting that non-AMI-CS is a risk factor for long-term mortality. AMI-CS was an independent risk factor predicting CV rehospitalization. Risk stratification targeting different outcomes and timings is warranted, and requires further investigation.

**Central Fig:**
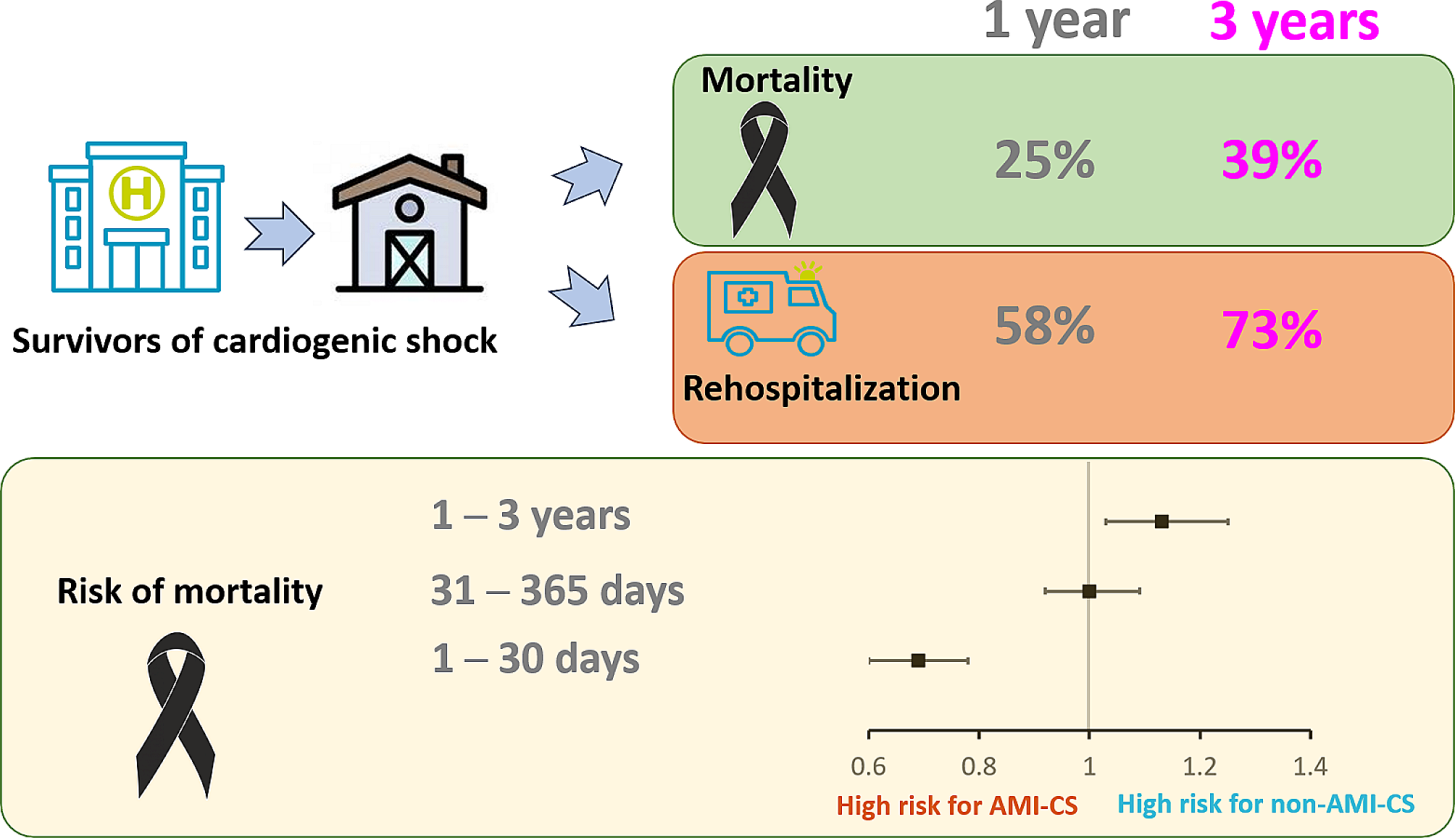
Mortality and rehospitalization among in-hospital survivors of CS are considerably high, with reports of 39% and 73% in 3 years. AMI-CS has time-varying prognostic effects. Patients with AMI-CS were associated with higher 30-day mortality, while such associations were attenuated and even reversed after a 1-year follow-up, suggesting that non-AMI-CS is a risk factor for long-term mortality. Abbreviation: AMI: acute myocardial infarction, CS: cardiogenic shock

### Electronic supplementary material

Below is the link to the electronic supplementary material.

Supplementary Material 1

Supplementary Material 2

Supplementary Material 3

Supplementary Material 4

## Data Availability

This study used the National Health Insurance Research Database (NHIRD), a healthcare administrative claims data source provided by the Health and Welfare Data Center (HWDC) of Taiwan’s Ministry of Health and Welfare. The HWDC is a third-party organization to which researchers can submit applications for access to health-related databases. Owing to legal restrictions imposed by the government of Taiwan under the Personal Information Protection Act, data cannot be made publicly available. All HWDC data were fully anonymized before access was acquired. In addition, these data can only be accessed and analyzed in an independent operating area in the HWDC, and only statistical results can be released from that operational area. Therefore, the original data cannot be shared publicly, owing to legal restrictions.

## Abbreviations

CS: Cardiogenic shock
AMI: Acute myocardial infarction
AMI-CS: Acute myocardial infarction-associated cardiogenic shock
IABP: Intra-aortic balloon pump
VAD: Ventricular assist device
MCS: Mechanical circulatory support
NHI: National Health Insurance
NHIRD: National Health Insurance Research Database
ICD-9-CM: International Classification of Diseases
Ninth Revision: Clinical Modification
NDR: National Death Registry
HR: Hazard ratio
CI: Confidence interval
PCI: Percutaneous coronary intervention
CABG: Coronary artery bypass graft
ECMO: Extracorporeal membrane oxygenation
