# Supplementary material for: Comparison of the prognosis among in-hospital survivors of cardiogenic shock based on etiology: AMI and Non-AMI
Source: Ann Intensive Care. 2024 May 12;14:74. doi: 10.1186/s13613-024-01305-2 (PMC11089020; doi:10.1186/s13613-024-01305-2)
Supplement: Supplementary file 1 — Supplementary Material 1 [file 13613_2024_1305_MOESM1_ESM.docx]

**Figure S1. The prevalence of hospital survivors of cardiogenic shock.** There is a rising trend of hospital survivors of cardiogenic shock from 2011 to 2017, p for trend < 0.001


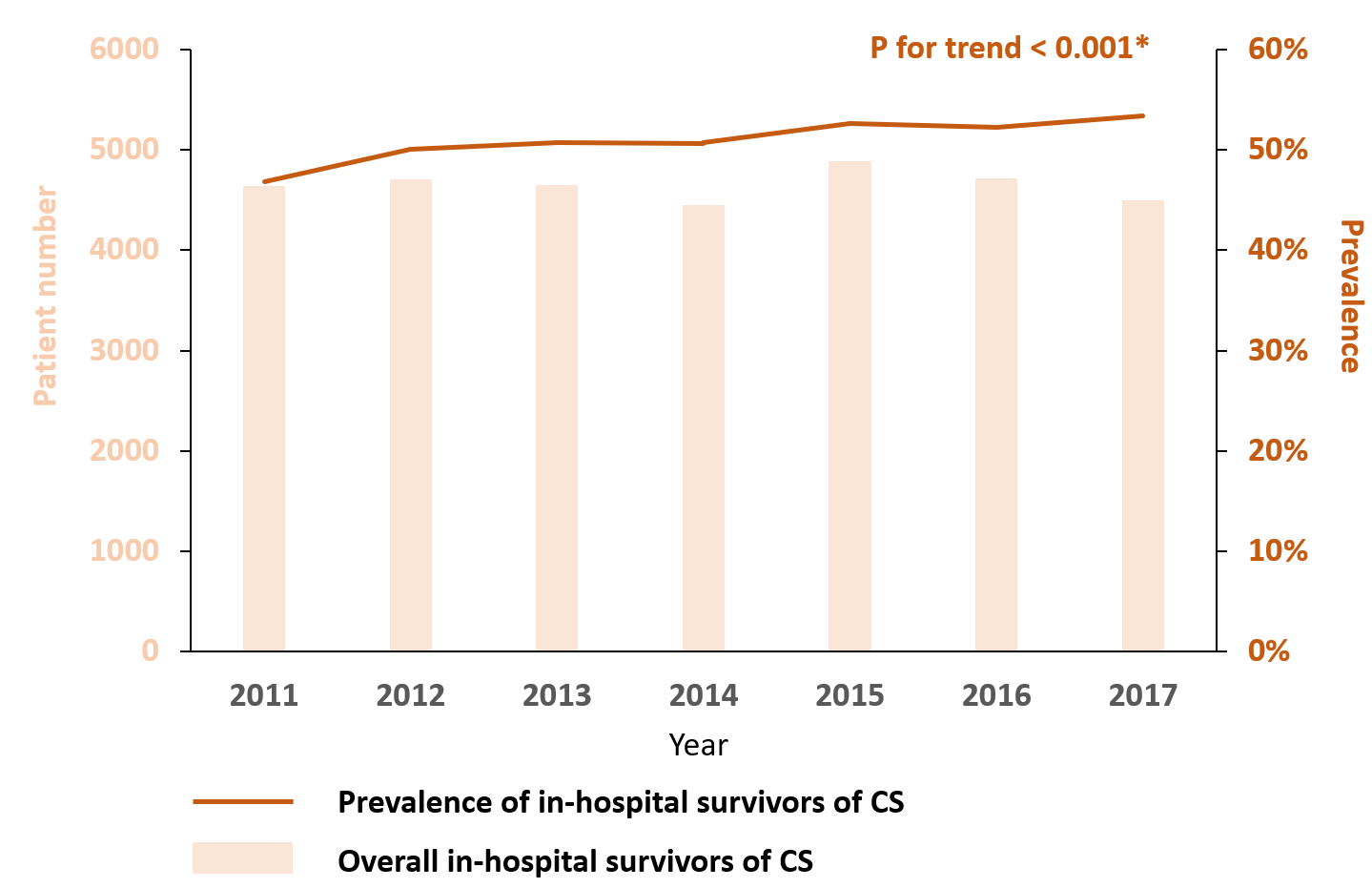


* P< 0.05

Abbreviation: AMI: acute myocardial infarction, CS: cardiogenic shock.
