# Supplementary material for: Comparison of the prognosis among in-hospital survivors of cardiogenic shock based on etiology: AMI and Non-AMI
Source: Ann Intensive Care. 2024 May 12;14:74. doi: 10.1186/s13613-024-01305-2 (PMC11089020; doi:10.1186/s13613-024-01305-2)
Supplement: Supplementary file 2 — Supplementary Material 2 [file 13613_2024_1305_MOESM2_ESM.docx]

**Table S1. Disease diagnostic coding, procedure coding and ATC code for medication**

| **Diseases / Comorbidity** | **ICD-9-CM / ATC** | **ICD-10-CM** |
| --- | --- | --- |
| Cardiogenic shock | 785.51 | R570 |
| Congestive heart failure | 428 | I50 |
| Hypertension | 401-405 | I10-I13, I15 |
| Diabetes mellitus | 250 | E08-E13 |
| Peripheral arterial disease | 440.2–440.4, 443.9 | I70, I73.9 |
| Dyslipidemia | 272 | E75, E77-E78 |
| Coronary artery disease | 413–414, 429.2 | I20, I25 |
| Myocardial infarction admission | 410 | I21-I22 |
| Renal failure | Certification in the Registry for Catastrophic Illness Patient Database | Certification in the Registry for Catastrophic Illness Patient Database |
| Stroke | 430-438 | G45-G46, I60-I63, I65-I69 |
| Malignancy | 140-239 | C00-C96, D00-D49, E31.22, J84.81, J91.0, K31.7, K63.5, Q85.0 |
| Atrial fibrillation | 427.3 | I48 |
| Cardiac arrest | Cardiac arrest: 427.5  Cardiac complications (0-4): 668.1  Cardiac complications:997.1 Sudden cardiac arrest: V12.53 | Cardiac arrest: I46.2, I46.8, I46.9 Cardiac complications (0-4): O74.2, O89.1 Cardiac complications:I97.7, I97.8 Sudden cardiac arrest: Z86.74 |
| **Procedure** |  |  |
| Percutaneous coronary intervention | 33076A, 33077A, 33078A, 33076B, 33077B, 33078B |  |
| Coronary artery bypass graft | 68023A, 68024A, 68025A, 68023B, 68024B, 68025B |  |
| Heart transplantation | 68035A, 68035B |  |
| Intra-aortic balloon pump | 33079A, 33079B |  |
| Extracorporeal membrane oxygenation | 68036A, 68036B |  |
| Ventricular assist device | 68051B |  |
| Cardiopulmonary resuscitation | 47029C |  |
| Hospice palliative care (NHID code) | 03001KB、. 03002AB、03003BB、03004BB, 05601K, 05602A, 05603B, 05605A, 05606B, P1101K, P11012A, P11013B, P1104K,P11015A, P11016B, P4401B, P4402B, P4403B | 03001KB、. 03002AB、03003BB、03004BB, 05601K, 05602A, 05603B, 05605A, 05606B, P1101K, P11012A, P11013B, P1104K,P11015A, P11016B, P4401B, P4402B, P4403B |
