# Supplementary material for: Comparison of the prognosis among in-hospital survivors of cardiogenic shock based on etiology: AMI and Non-AMI
Source: Ann Intensive Care. 2024 May 12;14:74. doi: 10.1186/s13613-024-01305-2 (PMC11089020; doi:10.1186/s13613-024-01305-2)
Supplement: Supplementary file 3 — Supplementary Material 3 [file 13613_2024_1305_MOESM3_ESM.docx]

**sTable 2. Hospice care after hospital discharge of cardiogenic shock**

|  | Overall | AMI | Non-AMI |
| --- | --- | --- | --- |
|  | *N = 16582* | *N* = 7,037 | *N* = 9,545 |
| Hospice care, n (%) | 3203 (19.3) | 1040 (14.8) | 2163 (22.7) |

Abbreviations: AMI: acute myocardial infarction.
