# Supplementary figures and images for: Comparison of the prognosis among in-hospital survivors of cardiogenic shock based on etiology: AMI and Non-AMI
Source: Ann Intensive Care. 2024 May 12;14:74. doi: 10.1186/s13613-024-01305-2 (PMC11089020; doi:10.1186/s13613-024-01305-2)

**Figure S2. Cause of death based on first International Classification of Diseases code.**


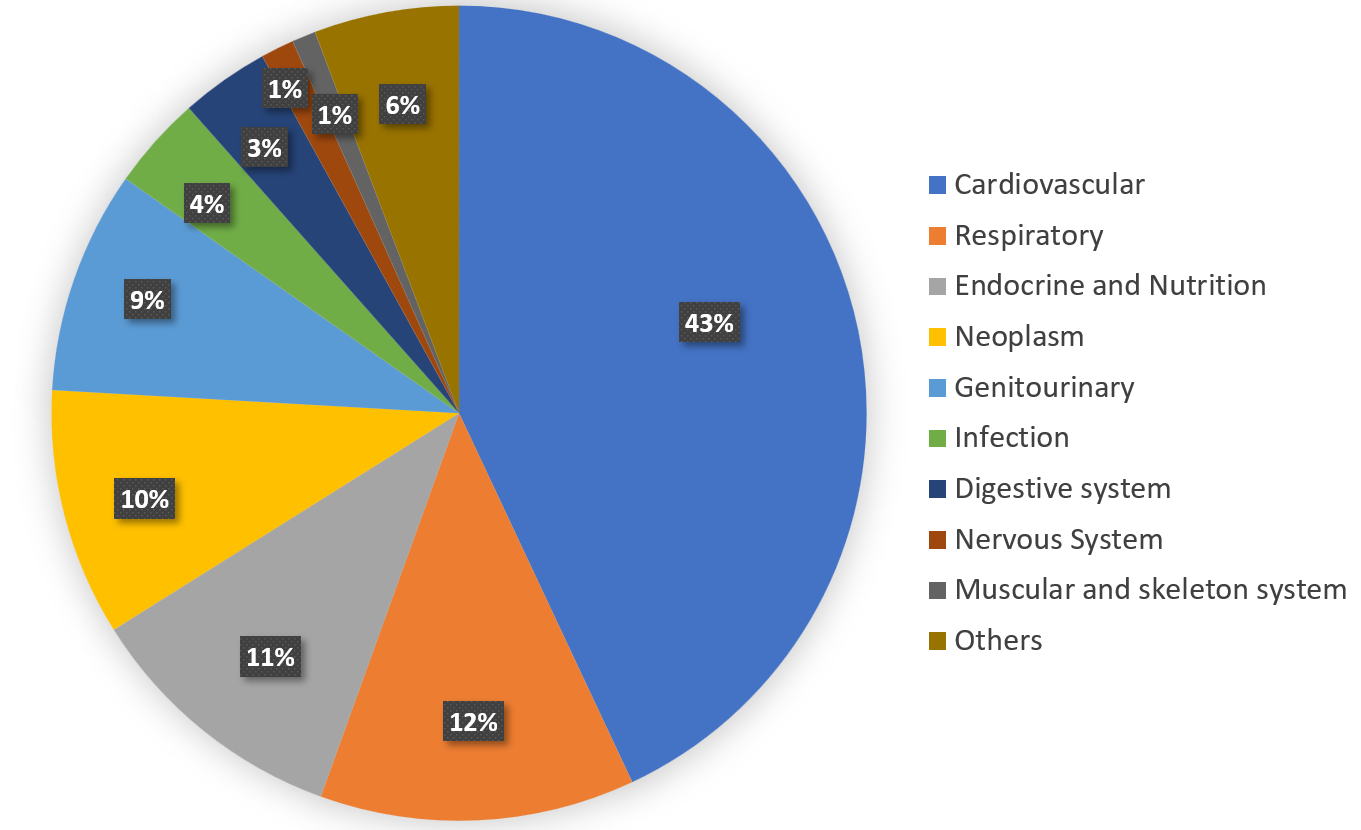

Supplement: Supplementary file 4 — Supplementary Material 4 [file 13613_2024_1305_MOESM4_ESM.docx]
